# Supplementary material for: Validity and reliability of a physical literacy knowledge, attitudes, self-efficacy and behaviors questionnaire for early childhood educators (PLKASB-ECE): An exploratory factor analysis
Source: PLoS One. 2024 Oct 28;19(10):e0312736. doi: 10.1371/journal.pone.0312736 (PMC11516012; doi:10.1371/journal.pone.0312736)
Supplement: S1 Appendix — (PDF) [file pone.0312736.s001.pdf]

**S1 Appendix.**

## Content validity of items on the PLKASB-ECE

| Final Round | Expert rating (3 or 4) | I-CVI† | Clarity* |
|---|---|---|---|
| I would rate my understanding of physical literacy as | 3 | 1.00 | 0 |
| Which of the following are key factors for physical literacy? Motivation | 3 | 1.00 | 0 |
| Which of the following are key factors for physical literacy? Genetics | 3 | 1.00 | 0 |
| Which of the following are key factors for physical literacy? Knowledge | 3 | 1.00 | 0 |
| Which of the following are key factors for physical literacy? Physical competence | 3 | 1.00 | 0 |
| Grasping would be an example of what movement skill? | 3 | 1.00 | 0 |
| What are the recommended minutes each day of physical activity for children 4 years old? | 3 | 1.00 | 0 |
| What percent of daily physical activity time for children age 4 years old should be child-led play | 3 | 1.00 | 0 |
| I understand what physical competence means. | 3 | 1.00 | 0 |
| I know what fundamental movement skills are. | 3 | 1.00 | 0 |
| Working on physical literacy in the early years sets a child up for being a physically active adult. | 3 | 1.00 | 0 |
| I feel confident including movement skills in my daily practice. | 3 | 1.00 | 0 |
| I feel confident identifying when a child is struggling with a movement skill. | 3 | 1.00 | 0 |
| I feel confident giving children strategies to help them improve their movement skills. | 3 | 1.00 | 0 |
| I plan 2 or more INDOOR physical activity experiences each day for children at my centre. | 3 | 1.00 | 0 |
| I plan 2 or more OUTDOOR physical activity experiences each day for children at my centre. | 3 | 1.00 | 0 |
| I ask the children to tell me what they are learning when they engage in physical activity. | 3 | 1.00 | 0 |
| I support children to be physically active in our centre. | 3 | 1.00 | 0 |
| These are examples of ways I support children to be physically active at our centre. | 3 | 1.00 | 0 |
| I do 30 minutes of heart pumping physical activity ___ days per week. | 3 | 1.00 | 0 |
| When planning my day I think about how I will find time to include physical activity for myself. | 3 | 1.00 | 0 |
| As a child, I was physically active outside of school time. | 3 | 1.00 | 0 |
| I enjoy being physically active. | 3 | 1.00 | 0 |
| Building my physical literacy skills can support my personal health throughout my life. | 3 | 1.00 | 0 |
| To me physical activity means... | 3 | 1.00 | 0 |

† The item content validity index (I-CVI) is calculated on item importance by dividing the number of experts issuing a judgment of “quite relevant” or “highly relevant” divided by the total number of experts. *Clarity where 0 = ‘as is’ or 1 = ‘rewording required’ by at least one rater.

| **Round 1 - Items** | **Expert rating (3 or 4)** | **I-CVI†** | **Clarity*** |
|---|---|---|---|
| I would rate my understanding of physical literacy as: | 2 | 0.67 | 0 |
| Motivation, confidence, knowledge and physical competence are all important in developing physical literacy | 2 | 0.67 | 1 |
| Grasping would be an example of what movement skill? | 3 | 1.00 | 0 |
| How many minutes of physical activity is recommended for children 4 years of age? | 2 | 0.67 | 1 |
| What percent of time should child led play contribute to physical activity for children age 4 years of age? | 3 | 1.00 | 0 |
| I understand what physical competence means. | 3 | 1.00 | 1 |
| I know what fundamental movement skills are. | 2 | 0.67 | 0 |
| I feel confident in my ability to incorporate fundamental movement skills in my early childhood professional practice. | 3 | 1.00 | 1 |
| I feel confident in my ability to identify when a child is struggling with performing movement skills. | 3 | 1.00 | 1 |
| I feel confident in my ability to give a child strategies to help them improve their movement skills. | 3 | 1.00 | 0 |
| I plan lots of different physical activity experiences inside and outside for children in our childcare centre | 3 | 1.00 | 1 |
| I encourage children to tell me what they are learning when they participate in physical activity. | 3 | 1.00 | 1 |
| I encourage children to tell me what they are learning when they participate in physical activity. | 3 | 1.00 | 1 |
| I encourage children to be physically active in our childcare centre. | 3 | 1.00 | 1 |
| How many days per week do you participate in 30 minutes of heart pumping physical activity? | 3 | 1.00 | 1 |
| When planning my day I think about how I will find time to include physical activity for myself. | 3 | 1.00 | 0 |
| As a child I participated in physical activity experiences outside of school regularly. | 2 | 0.67 | 0 |
| I enjoy participating in physical activity. | 2 | 0.67 | 0 |
| To me physical activity means... | 3 | 1.00 | 0 |

† The item content validity index (I-CVI) is calculated on item importance by dividing the number of experts issuing a judgment of “quite relevant” or “highly relevant” divided by the total number of experts. *Clarity where 0 = ‘as is’ or 1 = ‘rewording required’ by at least one rater.
