# Supplementary material for: Validity and reliability of a physical literacy knowledge, attitudes, self-efficacy and behaviors questionnaire for early childhood educators (PLKASB-ECE): An exploratory factor analysis
Source: PLoS One. 2024 Oct 28;19(10):e0312736. doi: 10.1371/journal.pone.0312736 (PMC11516012; doi:10.1371/journal.pone.0312736)
Supplement: S2 Appendix — (PDF) [file pone.0312736.s002.pdf]

S2 Appendix.

The Physical Literacy Knowledge, Attitudes, Self-Efficacy, and Behaviors Questionnaire for Early Childhood Educators

The purpose of this questionnaire is to find out what you know and think about physical activity and physical literacy. In this survey, physical activity is any activity that makes your heart beat faster or makes you get out of breath some of the time. Physical literacy is the ability and desire to be physically active.

		Very limited	Limited	Neutral	Good	Very good
1.	I would rate my understanding of physical literacy as	o	o	o	o	o

Section 1: These questions are about what you know on this topic.

		Key Factor	Not a Key Factor	I don' t know
Which of the following are key factors for physical literacy?				
2.	Motivation	o	o	o
3.	Genetics	o	o	o
4.	Knowledge	o	o	o
5.	Physical competence	o	o	o

6. Grasping would be an example of what movement skill?
   - o Locomotor
   - o Stability
   - o Object control
   - o I don't know

7. What are the recommended minutes each day of physical activity for children 4 years old?

30	40	50	60	70	80	90	100	110	120	130	140	150	160	170	180	190	200

8. What percent of daily physical activity time for children age 4 years old should be child-led play?

10	20	30	40	50	60	70	80	90	100

Section 2: These next questions are about your work at your child care centre.

		Strongly disagree	Disagree	Somewhat Disagree	Neutral	Somewhat Agree	Agree	Strongly Agree
9.	I understand what physical competence means.	o	o	o	o	o	o	o
10.	I know what fundamental movement skills are.	o	o	o	o	o	o	o
11.	Working on physical literacy in the early years sets a child up for being a physically active adult.	o	o	o	o	o	o	o
12.	I feel confident including movement skills in my daily practice.	o	o	o	o	o	o	o
13.	I feel confident identifying when a child is struggling with a movement skill.	o	o	o	o	o	o	o
14.	I feel confident giving children strategies to help them improve their movement skills.	o	o	o	o	o	o	o
15.	I plan 2 or more INDOOR physical activity experiences each day for children at my centre.	o	o	o	o	o	o	o
16.	I plan 2 or more OUTDOOR physical activity experiences each day for children at my centre.	o	o	o	o	o	o	o
17.	I ask the children to tell me what they are learning when they engage in physical activity.	o	o	o	o	o	o	o
18.	I support children to be physically active in our centre.	o	o	o	o	o	o	o

19. These are examples of ways I support children to be physically active at our centre.

Section 3: These next questions are about you.

20. I do 30 minutes of heart pumping physical activity ___ days per week?

0	1	2	3	4	5	6	7

		Strongly disagree	Disagree	Somewhat Disagree	Neutral	Somewhat Agree	Agree	Strongly Agree
21.	When planning my day, I think about how I will find time to include physical activity for myself.	o	o	o	o	o	o	o
22.	I enjoy being physically active.	o	o	o	o	o	o	o
23	Building my physical literacy skills can support my personal health throughout my life.	o	o	o	o	o	o	o
24.	As a child, I was physically active outside of school time.	o	o	o	o	o	o	o

25. To me physical activity means …
