# Supplementary material for: Validity and reliability of a physical literacy knowledge, attitudes, self-efficacy and behaviors questionnaire for early childhood educators (PLKASB-ECE): An exploratory factor analysis
Source: PLoS One. 2024 Oct 28;19(10):e0312736. doi: 10.1371/journal.pone.0312736 (PMC11516012; doi:10.1371/journal.pone.0312736)
Supplement: Attachment — Submitted filename: Response to Reviewers.docx [file pone.0312736.s003.docx]

PONE-D-24-06642

**Development and validation of a Physical Literacy Knowledge, Attitudes, Self-Efficacy, and Behaviors Questionnaire for Early Childhood Educators (PLKASB-ECE): An Exploratory Factor Analysis**

PLOS ONE

Thank you for offering us an opportunity to revise our paper *“Validity and reliability of a physical literacy knowledge, attitudes, self-efficacy and behaviors questionnaire for early childhood educators (PLKASB-ECE): An exploratory factor analysis”* (PONE-D-24-06642). We have included a response to reviewers in which we address each comment the reviewers made. The remarks of the reviewers are numbered and depicted first, followed by our responses to these comments. Corresponding changes are highlighted in the manuscript text.

Reviewer #1: ID: PONE-D-24-06642

**Overall**

**Reviewer #1 Comment #1:** The study includes both development and validation components, and the process of “validation of the questionnaire” should be emphasized in the title, in addition to the relevant description of the questionnaire development.

**Author Response (R1-C1):** Thank you for this suggestion. It is an important element of our study and we have edited the title as suggested. The title now reads *“Validity and reliability of a physical literacy knowledge, attitudes, self-efficacy and behaviors questionnaire for early childhood educators (PLKASB-ECE): An exploratory factor analysis”*

**Abstract**

**Reviewer #1 Comment #2:** Overall, as an Abstract, there are issues of length and redundancy, and the authors are advised to further minimize unnecessary descriptions.

**Author Response (R1-C2):** We have reviewed the abstract with this suggestion in mind. We revised the abstract to remove redundancies and clarified the details of the items in the revised EFA. These changes are reflected in the track changes manuscript.

**Reviewer #1 Comment #3:** Line 24-25, Page 2: “subsequently underwent several rounds of expert content validation……”, write in detail the number of rounds for which expert content validation is performed; vague terms are not recommended.
**Author Response (R1-C3):** We agree. We have revised the sentence with specific details and Line 31 now reads *“subsequently underwent three rounds of expert content validation……”*

**Reviewer #1 Comment #4:** Line 28-29, Page 2: The time period for validation sample collection was? Please add any relevant information.
**Author Response (R1-C4):** Thank you for catching this detail. We have aligned the time period for sample collection with the methods section. Line 35-36 now reads: *“The PLKASB-ECE tool was administered to 470 educators in Alberta, Canada between 2019 to 2022.”*

**Reviewer #1 Comment #5:** Overall: “PLKASB-ECE”, is this an abbreviated form of the scale? Didn't find an explanation of the full name in the Abstract.
**Author Response (R1-C5):** Thank you for identifying this. We have changed the abstract ordering of words and addition of the acronym to better align with the name of the tool. Line 26 now reads: *“The purpose of this study was to develop a holistic digital tool to measure physical literacy knowledge, attitudes, self-efficacy, and behaviors of early childhood educator (PLKASB-ECE) for both their professional teaching context as well as the educator’s own personal context.”*

**Reviewer #1 Comment #6:** Keywords: Quantitatively, there are too many keywords set, and it is recommended that some that are not relevant to the focus of the study be removed.

**Author Response (R1-C6):** We have removed keyword sets and there are now 5 keyword sets

**Introduction**

**Reviewer #1 Comment #7:** Line 38, Page 2: How does the author define “physical activity” before describing the phenomenon in question?
**Author Response (R1-C7):** Thank you for this suggestion. We have included a definition at the beginning of the introduction to establish context. Line 44 now reads: *“Physical activity, bodily movement resulting from skeletal muscle activation leading to increased energy expenditure, has been identified as a protective factor for human health”*

**Reviewer #1 Comment #8:** Line 47-56, Page 3: Are the disadvantages of physical inactivity or the benefits of physical activity specific to the group of childhood aged 0-5 years? It is recommended that the authors add relevant statements, which will help to reflect the need to study the population.
**Author Response (R1-C8):** We agree. We have reorganized the introduction to bring the paragraph of the health benefits of physical activity into the first paragraph and included the disadvantages of physical inactivity related to NCDs in this first paragraph. Line 51-56 now provide both the benefits of physical activity and disadvantages of physical inactivity.

**Reviewer #1 Comment #9:** Line 62-64, Page 3: Elaborating on the mechanisms by which these four interrelated domains (affective, physical, cognitive, behavioral) contribute to the holistic development of physical literacy facilitates a clearer presentation and is recommended to be added by the authors.
**Author Response (R1-C9):** Thank you for this suggestion. We have added the following to better describe this important concept: Line 93 *“This can be conceptualized in a framework of four interrelated domains (affective, physical, cognitive, behavioural) that contribute to the holistic development of physical literacy that consider the social processes associated with lifelong learning and prioritization in lifelong physical activity engagement (Cairney et al., 2019; Cornish et al., 2020). It has been proposed that a strong physical literacy foundation will improve an individual’s overall health and wellness through the building of an individual’s positive relationship with daily movement (Cornish et al., 2020; Edwards et al., 2017; Shearer et al., 2021; Wainwright et al., 2020).*

**Reviewer #1 Comment #10:** Line 64-66, Page 3: More presentations of relevant mechanisms, or more detailed descriptions are necessary.
**Author Response (R1-C10):** We have improved the connections and details as described above (R1-C9).

**Reviewer #1 Comment #11:** Line 71-73, Page 4: “These settings provide a fertile ground for targeting strategies for nurturing physical literacy in the early years”, how do these environmental factors affect the quality of education? Related content suggestions are presented.
**Author Response (R1-C11):** Thank you for this comment. With the restricting of the introduction to improve flow we connect physical activity to improved health outcomes that can be taught through a physical literacy lens that is provided in high-quality child care environments. Line 96 now reads: “*High quality child care has long-term effects on a child’s cognitive, academic, and language development tracking into adolescence (Vandell et al., 2010). Early childhood educators’ health knowledge and practices contribute to high quality care and are vital in creating a health-supportive environment, influencing a child's lifelong relationship with physical activity (Lugossy et al., 2022; World Health Organization, 2016).”*

**Reviewer #1 Comment #12a:** Line 76, Page 4: What does “ECEC” mean? Line 73-79, Page 4: Is the professional preparation of early childhood educators only about education? The relevant descriptions are limited.
**Author Response (R1-C12):** ECEC is defined earlier in the manuscript, but only in the abstract. We have added the full written component to the Introduction: Line 79-80 now reads “*early childhood education and care (ECEC) settings”*

**Reviewer #1 Comment #12b:** Line 73-79, Page 4: Is the professional preparation of early childhood educators only about education? The relevant descriptions are limited.

**Author Response (R1-C12b):** Thank you for pointing this out. The descriptions of the education preparation of educators require clarity. We have revised the description for clarity - Line 101-103 **“…** *some educators have completed an early childhood education certificate after completing high school whereas others report having college degrees with early childhood education diplomas.”* This description aligns with demographic descriptions presented in Table 1.

**Reviewer #1 Comment #13:** Line 92-93, Page 5: “In practice, very little time is available during the workday for early childhood educators to pursue this task”, is there a basis for supporting this view in detail?
**Author Response (R1-C13):** Thank you for the clarification request. We see this in our work regularly and it is further outlined in the literature. We have added three references to support educators’ time limitation barrier to professional development. Line 127-128 now reads *“In practice, very little time is available during the workday for early childhood educators to pursue this task (McKinlay et al., 2018; Brussoni et al., 2021; Pölzl-Stefanec, 2021).”*

**Reviewer #1 Comment #14:** Line 95-100, Page 5: The representation of relevant measures is inadequate, and the importance of a brief and valid assessment tool for physical literacy for early childhood educators has not been adequately captured.
**Author Response (R1-C14):** We have included an additional systematic review looking for assessment tools in this area that concludes there is a paucity of tools available for assessing early childhood educators as a proxy for the child care setting in promoting physical activity in the young child. We add this article to provide the rationale for a brief and valid assessment tool for physical literacy for early childhood educators. Line 132-135 now reads: *“In another systematic review it was noted that there was limited research for assessment tools reporting validity and reliability measures available to measure early childhood educators’ self-efficacy in supporting young children’s physical activity and reduction in sedentary behaviour (Szpunar et al., 2021).”*

**Reviewer #1 Comment #15:** Line 99, Page 5: “Self-efficacy” is not an unusual term and it is suggested that a definition be added.
**Author Response (R1-C15):** We have included a definition to provide more clarity on the use of ‘self-efficacy’ within a social cognitive theory framework by adding Line 115-121 *“Social cognitive factors have been used to understand why people engage in certain behaviors and are believed to predict intentional behavior (Ajzen, 1991; Bandura, 2004; De Vries, 2017; Janz & Becker, 1984). Social cognitions include constructs such as attitude and efficacy beliefs. Attitude is defined as the perceived favorable or unfavorable evaluation of a certain behavior (Ajzen & Fishbein, 2002), while self-efficacy refers to the belief in one’s ability to perform a particular behavior (Bandura, 1977). In the context of physical literacy and early childhood education, research suggests that efficacy beliefs of educators can influence their ability and intention to facilitate movement for children (Szpunar et al., 2021; Copeland et al., 2012).”*

**Reviewer #1 Comment #16:** Overall: There is too little about how your study compares to other similar studies to highlight the innovative nature of your study, and additions are recommended.

**Author Response (R1-C16):** Thank you for suggesting we highlight the innovative nature of our study. We have provided context with the changes described in Comment #14. This is also explored in great detail in Phase 1 of the results section as it is part of the needs assessment portion of the methods. We also address this in the discussion section when contextualizing the PLKASB-ECE in the context of the literature that exists currently.

**Methods**

**Reviewer #1 Comment #17:** Line 105, Page 5: The setting of subheading case should be standardized throughout the text, please note and revise the relevant sections.

**Author Response (R1-C17):** Thank you for identifying this. We have revised the headings to be standardized throughout the manuscript.

**Reviewer #1 Comment #18:** Line 110-112, Page 5 ＆ Line 113-124, Page 6: Does the search keywords include only “physical literacy”, without considering other similar terms?
**Author Response (R1-C18):** The search term used was “physical literacy” along with truncated versions such as “physical lit*” to focus the search on physical literacy concepts and avoid confusion with ‘play’ or ‘physical activity’. This structure is similar to search strategies used in Physical Literacy Systematic Reviews conducted by Edwards et al., 2018; Jean de Dieu & Zhou, 2021; Carl et al., 2022.

- Jean de Dieu, H., & Zhou, K. (2021). Physical literacy assessment tools: a systematic literature review for why, what, who, and how. *International Journal of Environmental Research and Public Health*, *18*(15), 7954.
- Carl, J., Barratt, J., Toepfer, C., Cairney, J., & Pfeifer, K. (2022). How are physical literacy interventions conceptualized?–a systematic review on intervention design and content. *Psychology of Sport and Exercise*, *58*, 102091.
- Edwards, L. C., Bryant, A. S., Keegan, R. J., Morgan, K., Cooper, S. M., & Jones, A. M. (2018). ‘Measuring’ physical literacy and related constructs: A systematic review of empirical findings. *Sports Medicine*, *48*, 659-682.

**Reviewer #1 Comment #19:** Line 117-121, Page 6: An introduction to ECEC content would be more appropriately placed in a separate paragraph of the introduction.

**Author Response (R1-C19):** Thank you for this observation. We have moved the ECEC content from page 6 to the Introduction. See Line 110 - 121

**Reviewer #1 Comment #20:** Face validity, Page 7: Please specifically describe the assessment process, criteria, and the handling of the results.

**Author Response (R1-C20):** We have changed the sentence for clarity. Line 171-175 now reads *“To address face validity of the scale, two in-service ECE educators, two pre-service ECE students, and two ECE university faculty members were identified for their expertise to provide a stakeholder perspective.* *The survey questions were provided to individuals to read and the research team took notes of verbal feedback regarding item clarity, comprehension, and suitability to the community.”*

**Reviewer #1 Comment #21:** Line 144-146, Page 7: Please add the treatment when evaluating readability, when F-K and FRE show consistent or discrepant results.

**Author Response (R1-C21):** The readability score report from Microsoft Word software is provided in a single report. The equations to compute readability are incorporated into versions of Microsoft Word and Zhou et al., (2017) suggest using Microsoft Word when Grade Level is needed as it makes the fewest error in linguistic elements. We have added the following for clarity Line 179-180: *“The F-K score was the primary outcome in evaluating readability.”* [Zhou, S., Jeong, H., & Green, P. A. (2017). How consistent are the best-known readability equations in estimating the readability of design standards? IEEE Transactions on Professional Communication, 60(1), 97-111.]

**Reviewer #1 Comment #22:** Line 156-158, Page 7 ＆ Line 159-160, Page 8: The number of ECEC centers selected and their proportion in large urban population centers, medium population population centers and low population centers should likewise be carefully accounted for.

**Author Response (R1-C22):** Thank you for this comment. We intended to communicate our effort to recruit child care centers throughout the province and with representation in various size communities. We have added a sentence to reflect the effort of this recruitment strategy. Line 195--198 now reads: *“Large urban population centers represent approximately 70% of the provinces population with 16% and 13% falling in the medium and small population centers, respectively (Government of Canada, 2017). We looked to balance the proportion of center recruitment relative to the overall provincial population.”*

**Reviewer #1 Comment #23:** Line 160-163, Page 8: “In the first year of data collection, center directors were invited to participate through phone correspondence, provided with general information on the study and allocated to either intervention or control group based on center directors’ assessment of both available time and capacity.” Please tell us what the criteria were for the center director to be assigned to the intervention or control group.

**Author Response (R1-C23):** All data for this study is derived from baseline testing before intervention. Concerning allocation, the center directors could assess their center staff’s time and capacity to enroll in the intervention component or control group. Line 208-211 *“In the first year of data collection, center directors were invited to participate through phone correspondence, provided with general information on the study and allocated to either intervention or control group based on center directors’ assessment of both available time and capacity.”*

**Reviewer #1 Comment #24:** Line 160-171, Page 8: The “Participants” section is overly oriented towards the screening of ECEC centers. Are there clear inclusion and exclusion criteria for participants?

**Author Response (R1-C24):** The target participant is an early childhood educator (classroom educator or director) employed in an ECEC center. We have added the classroom educator or director to the sentence to clarify this. Line 207-209 now reads: *“Each participating director was requested to identify three staff members (classroom educator or director) to be included in the study, with a preference to include two educators and the center director.”*

**Reviewer #1 Comment #25:** Line 169-171, Page 8: “Each participating center was requested to identify three staff members to be included in the study, with a preference to include two educators and the center director or manager.” How were these two educators chosen? Also, how are they elected if there is more than one center director or manager in the participating centers?

**Author Response (R1-C25):** The structure of ECEC centers is to have one director decision-maker for the unit. There can be assistant directors who provide leadership support to the director; however, the director is the lead for the center. In our work, we spoke and coordinated with the director as they understand their organizational unit and determine job assignments as part of their role title. We have clarified this by replacing ‘center’ with ‘director’ and removing the term manager for clarity. Line 207-209 now reads: *“Each participating director was requested to identify three staff members (classroom educator or director) to be included in the study, with a preference to include two educators and the center director.”*

**Reviewer #1 Comment #26:** Line 184-186, Page 9: “In exploratory factor analysis a recommended minimum subject-to-item (N:p) ratio ranges from 5:1 to 10:1” How do you determine that this is the optimal ratio? Please add supporting literature.

**Author Response (R1-C26):** Thank you for this question. Tinsley and Tinsley (1987) suggest 5 to 10 participants (N) per item (p) with a N:p ratio of 5:1 as the low end and 10:1 at the high end as a sample size estimation for factor analysis. The authors suggest that after N>300 this ratio can be relaxed. Kyriazos (2018) also addresses sample size recommendations in his paper “Applied Psychometrics: Sample Size and Sample Power Considerations in Factor Analysis (EFA, CFA) and SEM in General”. Kyriazos suggests that in the end, the proof is in the “strength of the item loadings” and “number of items per factor” that speaks to the strength of the data. These two references are included as supporting literature on Line 225.

**Reviewer #1 Comment #27:** Page 8: Expressions related to ethical norms such as informed consent of participants are needed to be added.

**Author Response (R1-C27):** Thank you. We have added the following to the end of the participant paragraph. Line 259-260: *“All participants were given ample time to read and review the consent forms prior to participating in the research study.”*

**Results**

**Reviewer #1 Comment #28:** Participants, Page 12: “78.5% of these centers were from large urban population cities”, does this; distribution of centers lead to bias due to unevenness?

**Author Response (R1-C28):** Thank you for this comment. In reviewing the categories, it was found that the distribution was 357 (76.0%) large urban, 45 (9.6%) medium, 68 (14.5%) small population centers. This is reflected in Table 1. With the changes made based on comment #22, we can contextualize the proportion from the different geographical areas. As this is an interpretation, we have added this to the discussion section. Line 443-450 now reads: *“In addition, the recruited centers were representative of child care centers across a large geographical region and alignment with provincial population distribution. The recruited centers 76.0% large urban, 9.6% medium, and 14.5% small population centers were relatively similar to overall population distribution across the province of 70% large urban, 16% medium, and 13% small population centers [51].”*

**Reviewer #1 Comment #29:** Line 253-254, Page 12: “After recording and editing the readability of the final items ranged from 5.2 to 9.5.” I don't quite understand how this process is accomplished, please explain in detail.

**Author Response (R1-C29):** We assessed each questionnaire item’s F-K grade reading level using Microsoft Word software in addition to the overall questionnaire F-K grade reading level. Line 297-301 now reads: *“In the first version of the questionnaire, the Flesch-Kincaid Grade Level (F-K) score for each individual questionnaire item ranged from a grade 5.2 to 11.5 reading level. After suggested changes from physical literacy experts during the face validity feedback, the research team revised wording which resulted in an F-K score of Grade 5.2 to 9.5 reading level for each item.”*

**Reviewer #1 Comment #30:** Table 1, Page 13: Comparing the unbalanced sex ratio, the sample is very skewed towards males, is this similar to the overall distribution of genders in the group?

**Author Response (R1-C30):** In Table 1, women participants make up 97.7% of the participant group with men making up only 2.3%. We have addressed this concern in the limitation section – Line 461-463 reads: “*Second, most participants (97.7%) were women. However, women represent 96% of the ECEC workforce in Canada so this sampling is reflective of the intended population (50).”*

**Discussion**

**Reviewer #1 Comment #31:** Line 365-366, Page 22: “This tool addresses a gap that has been identified for the early childhood (i.e. 0-5) community while also trying to address a holistic measurement of physical literacy from both a qualitative and quantitative perspective.” All of your studies were of young children aged 2-5 years, and it seems inappropriate to say 0-5 years here.

**Author Response (R1-C31):** Thank you for pointing this out. We have corrected the statement to restrict to the 2-5 years age group. Line 420 now reads: *“This tool addresses a gap that has been identified in the early childhood (i.e., 2-5 years) community …”*

**Reviewer #1 Comment #32:** I have thoroughly reviewed the entire manuscript, and I must commend the authors for conducting an exhaustive study. The authors have undertaken a substantial amount of work, which is undeniable. Nevertheless, a number of issues remain to be noted, modified, added or deleted. I hope the authors will address the comments comprehensively in the final version, enhancing the depth and persuasiveness of the research.

**Author Response (R1-C32):** Thank you for your comments and suggestions. We believe the edits resulting from this feedback has improved the quality of the manuscript.

Reviewer #2:

**Reviewer #2 Comment #1:** Thank you for the opportunity to review the manuscript PONE-D-24-06642.e.
The study holds practical significance, but the analysis results are dubious for the following reasons: **Author Response (R2-C1):** Thank you for your comments. We believe the tool will be of value to community health research. We have worked to address your suggestions regarding the analysis.

**Reviewer #2 Comment #2:** The exploratory factor analysis (EFA) is incomplete. As per Table 3, the item "As a child, I was physically active outside of school time" lacks a satisfactory factor loading on any factor, indicating a weak correlation with other items. This item should be removed, followed by a revised EFA to assess if the remaining items still support a 5-factor model.
**Author Response (R2-C2):** Thank you for this comment. We have rerun the analysis without the non-loading items (#20 - *As a child, I was physically active outside of school time* and the summed knowledge score), and we found near equivalent results with this 5-factor solution. This is now reflected in Table 3.

**Reviewer #2 Comment #3:** Additionally, the factor structure of the Physical Literacy Knowledge, Attitudes, Self-Efficacy, and Behaviors Questionnaire has not been validated with a new sample using confirmatory factor analysis. Consequently, it's unclear if the 5-factor model is optimal, despite initial EFA findings suggesting its promise. The unresolved factor structure issue undermines the robustness of the study's conclusions and limits its applicability.
**Author Response (R2-C3):** We thank the reviewer for this suggestion. Scale development is an iterative and ongoing process. We have provided a detailed stepwise explanation of the development of the tool thus far. We have indicated in the manuscript that future research is to recruit a new independent sample of participants to conduct a CFA to confirm the factor structure. This approach is similar to other articles in the process of tool development: Marr, C., Vaportzis, E., Niechcial, M. A., Dewar, M., & Gow, A. J. (2021). Measuring activity engagement in old age: An exploratory factor analysis. *Plos one, 16*(12), e0260996. We have included this in the limitation section. Line 461-466 now reads: “*Lastly, the factor structure of the PLKASB-ECE has not been validated with a new independent sample using confirmatory factor analysis. The sample size in the current study was not sufficient to partition for dual exploratory and confirmatory factor analysis. The next step in the iterative process of scale development for PLKASB-ECE is to conduct confirmatory factor analysis with an independent sample to substantiate the factor structure indicated in this study.”*
