# Supplementary material for: Validity and reliability of a physical literacy knowledge, attitudes, self-efficacy and behaviors questionnaire for early childhood educators (PLKASB-ECE): An exploratory factor analysis
Source: PLoS One. 2024 Oct 28;19(10):e0312736. doi: 10.1371/journal.pone.0312736 (PMC11516012; doi:10.1371/journal.pone.0312736)
Supplement: Attachment — Submitted filename: Response to Reviewers.docx [file pone.0312736.s004.docx]

PONE-D-24-06642R1

**Validity and reliability of a physical literacy knowledge, attitudes, self-efficacy and behaviors questionnaire for early childhood educators (PLKASB-ECE): An exploratory factor analysis**

PLOS ONE

**Journal Requirements**

**Journal Requirements #1**

Any changes to the reference list should be mentioned in the rebuttal letter that accompanies your revised manuscript.

**Author Response (JR#1):** We have removed the following references from the revised manuscript. In response to Reviewer #3 Comment #3 we significantly revised Lines 128-149 to strengthen rationale for the scale and explain the originality of the tool and study. This revision resulted in the removal of the following citations and the addition of new references. The new references cited are in red in the revised manuscript with track changes.

- Brussoni et al. JMIR Res Protoc. 2021;10: e31041. doi:10.2196/31041
- McKinlay et al. Australas J Early Child. 2018;43: 32–42. doi:10.23965/AJEC.43.2.04
- Pölzl-Stefanec E. Br J Educ Technol. 2021;52: 2192–2208. doi:10.1111/bjet.13124
- Boldovskaia et al. PLOS ONE. 2023;18: e0288541. doi:10.1371/journal.pone.0288541

**Editor**

**Editor Comment #1**

I have one request. Can you make your headings the same font and font size as the other parts of the paper?

**Author Response (EC#1):** We have selected all text and made all text the same font (Times New Roman) and font size (12 pt). We have verified that the headings are the same font and font size as all text in the manuscript.

Reviewer #1: ID: PONE-D-24-06642R1

**Overall**

**Reviewer #1 Comment #1:** The author has made careful revisions and the quality of the article has been greatly improved, congratulations! However, some detailed issues still need to be improved.

**Author Response (R1-C1):** Thank you for the opportunity to revise this manuscript. We appreciate all the feedback and feel that the changes have improved the work. Please see our changes based on the additional suggestions.

**Abstract**

**Reviewer #1 Comment #2:** Overall, Subheadings can be used to separate the content of the abstract, including “Background”, “Objective”, “Methods”, “Results”, “Conclusion”, etc.

**Author Response (R1-C2):** Thank you for this suggestion. We have updated the abstract to include each subheading.

**Introduction**

**Reviewer #1 Comment #3:** Line 66-74, Page 4: Perhaps it would make more sense to present the situation of the group of early childhood aged 0-5 years in a separate paragraph.

**Author Response (R1-C3):** We have separated the paragraph to present the context of early childhood 0-5 year on its own (starting at Line 71).

**Methods**

**Reviewer #1 Comment #4:** Figure 1: It still looks blurry, is there a higher pixel version? Suggest the author to replace figure 1.

**Author Response (R1-C4):** Thank you for mentioning this. We have resubmitted the image at a higher resolution (300 dpi) 2250 × 1522 according to journal instructions.

**Results**

**Reviewer #1 Comment #5:** Table 1, Page 15: Table 1 seems to lack an explanation of abbreviations, both in the title and in the contents of the table.

**Author Response (R1-C5):** A notes section has been added to provide explanation of abbreviations in table 1 (Line 329 to 332).

**Reviewer #1 Comment #6:** Table 2, Page 17: The information given in the table is on the low side, would it be considered to move the content to be accounted for in the body of the text instead of setting up a table?

**Author Response (R1-C6):** While we could provide this information in the body of the text, having the information in a table makes the comparison between the four-factor model and the five factor model a quick read that provides the reader with information quickly. We suggest that keeping it in a table is a reader friendly decision and have left it as a table.

Reviewer #3: ID: PONE-D-24-06642R1

**Overall**

**Reviewer #3 Comment #1:** The authors are commended for developing and evaluating a tool to assess ECEs knowledge, self-efficacy and attitudes towards physical literacy in early learning environments.

**Author Response (R3-C1):** We appreciate the feedback.

**Reviewer #3 Comment #2:** Overall, I think that the manuscript could be greatly improved with more attention to the description of Physical Literacy (PL), and how this relates to and is distinct from Physical Activity (PL Consensus Statement, Tremblay et al., 2018).

**Author Response (R3-C2):** We have revised the introduction to provide a clearer distinction between PL and PA. This now reads.

*“Physical literacy encompasses a comprehensive approach to increasing physical activity participation which in turn supports child development [16]. While physical activity relates to bodily movement, Canada’s Physical Literacy Consensus Statement defines physical literacy as “the motivation, confidence, physical competence, knowledge, and understanding to value and take responsibility for engagement in physical activities throughout life” [17]. It addresses the lifelong journey of who, what, where, when, why, and how to cultivate the engagement in physical activity that enhances personal health. Physical literacy can be conceptualized in a framework of four interrelated domains: affective, physical, cognitive, and behavioral [17]. This holistic approach emphasizes the social processes associated with lifelong learning and sustained physical activity engagement [17–19].”*

**Reviewer #3 Comment #3:** Despite the revisions, the manuscript would benefit from a stronger rationale for the scale and how this could be used as a proxy to evaluate PL interventions aimed at children. The originality of this study is not communicated. Greater detail and description of the holistic nature of the scale, the components involved and what this means would improve readability.

**Author Response (R3-C3):** Thank you for this comment. We have significantly revised Lines 128-149 to strengthen rationale for the scale and explain the originality of the tool and study.

**Reviewer #3 Comment #4:** Finally, there remains too little discussion of how this study relates to others. For example, the discussion is very short and doesn’t capture all of the interesting elements of the data derived. The manuscript would be improved by providing more detailed discussion of similar studies to support the findings.

**Author Response (R3-C4):** We appreciate this comment. We have expanded the discussion section Lines 435 to 445 to capture some additional elements of the data derived. We have added information to the discussion section to provide context of our study to similar studies. See Lines 448 to 490.

**Abstract
Reviewer #3 Comment #5:** Line 23-24 – I suggest improving this sentence by identifying “this construct”, as well as the type of interventions (i.e., PL or PA) you are referring to and how ECEs relate to PL among children.

**Author Response (R3-C5):** We have revised the background section of the abstract (Lines 23-25) to address these three suggestions to *“However, enhancing ECEs’ ability to foster physical literacy requires valid and reliable assessment tools to measure holistic physical literacy constructs (i.e., cognitive, affective, behavioral) in order to assess the impact of physical literacy educational interventions.”*

**Reviewer #3 Comment #6:** Line 27 – What is meant by their “own personal context”?

**Author Response (R3-C6):** We are referring to the educator’s personal relationship with movement. This is relevant as personal affinity for movement has been found to be a predictor of providing more physical activity opportunities for children which is discussed in more detail in the Introduction paragraph 5. To improve the abstract, we have revised Line 29 to “*the educator’s own personal physical literacy behaviours.*”

**Reviewer #3 Comment #7:** Line 32 – The readability score of K-F Grade 8.7 was confusing until I read the manuscript. Consider adding more information here.

**Author Response (R3-C7):** Thank you for the suggestion. We have edited the sentence for clearer language, and provided the full name "Flesch-Kincaid Grade Level" as "K-F" might not be immediately recognizable to all readers. This sentence now reads “*Six additional items were added, resulting in a final 25-item self-report measure with a readability score equivalent to an 8th grade reading level (Flesch-Kincaid Grade Level: 8.7).”*

**Reviewer #3 Comment #8:** Line 41 – For clarity, I suggest including the type of intervention (e.g., PL among children).

**Author Response (R3-C8):** We have improved the clarity of the sentence to include the type of intervention (Line 34-35). The sentence now reads “*This tool will be valuable as a research outcome measure for assessing educational interventions aimed at enhancing educators’ understanding, confidence, and strategies for fostering holistic concepts of physical literacy in young children within ECECs*.”

**Methods
Reviewer #3 Comment #9:** Why were cooks/chefs included as participants? This seemed odd.

**Author Response (R3-C9):** In Table 1 we include the heading Position where Cook/Chef appears. In ECECs within the province of Alberta, all individuals who can be part of the education and care of the ECEC children must have a minimum of Level 1 education. Some Cook/Chefs have completed the Level 1 educator training and work with children when they are not working preparing meals. This means they engage with children directly in the various components of the day which includes aspects of physical activity (indoor/outdoor time). In this way they impact the physical literacy environment and need to be part of the population on which the tool has been validated. Not all centers have Cook/Chefs, not all Cook/Chefs have completed the Level 1 education, so the relatively low percentage of Cook/Chefs is representative of the provincial population. We hope that contextualizes cook/chef within this participant group.

**Reviewer #3 Comment #10:** Greater detail about how consent was obtained directly from the ECEs is important to include. The general statement at the end of this section does not effectively allow for replication or demonstrate procedures adequately.

**Author Response (R3-C10):** Thank you for pointing this out. We have provided more detail in the Methods section (Line 214-218) which now reads “*Centers that agreed to participate invited three educators from their center to join the research study. Each educator received a personal email containing: information about study participation, private secure digital access to their consent form, and contact information for a trained research associate to answer potential questions.*”
