# Supplementary material for: Validity and reliability of a physical literacy knowledge, attitudes, self-efficacy and behaviors questionnaire for early childhood educators (PLKASB-ECE): An exploratory factor analysis
Source: PLoS One. 2024 Oct 28;19(10):e0312736. doi: 10.1371/journal.pone.0312736 (PMC11516012; doi:10.1371/journal.pone.0312736)
Supplement: Attachment — Submitted filename: physical scale.docx [file pone.0312736.s005.docx]

Objective: The purpose of this study was to develop a holistic digital tool to measure physical

27 literacy knowledge, attitudes, self-efficacy, and behaviors of early childhood educators

28 (PLKASB-ECE) for both their professional teaching context as well as the educator’s own

A 78 systematic review of objectively measured physical activity in center-based child care revealed that 79 physical inactivity levels are high across worldwide geographical locations [13]

Exploring educator’s social cognitions can provide deeper insights 117 into how they can influence their behaviors in supporting the development of children’s physical 118 literacy. A complex set of factors govern educational decisions and behaviors within the ECEC 119 environment. The critical ecology framework [32] proposes that educator behaviors are 120 influenced by relationships and social interactions within the various contexts of an ECEC 121 environment. Social cognitive factors have been used to understand why people engage in certain 122 behaviors and are believed to predict intentional behavior [33–36]. Social cognitions include 123 constructs such as attitude and efficacy beliefs. Attitude is defined as the perceived favorable or 124 unfavorable evaluation of a certain behavior [37], while self-efficacy refers to the belief in one’s 125 ability to perform a particular behavior [38].

This section seems like a list of statements that is not supporting the paragraph it is in—please take it out

Six were review articles [63–68], twelve addressed kindergarten to grade 6 [69–76] or 286 grade 7 to 12 [18,77–79] school aged children motor skills measurement of physical literacy, one 287 article described the development of a physical literacy questionnaire for primary school teachers 14 288 [80], and one provided a qualitative assessment of physical literacy in primary school teachers 289 [81]. Please make these into three separate sentences

It consists of six subscales: 1) Knowledge sum score (n=7) 388 measuring educators’ physical literacy knowledge; 2) Perception of Knowledge (n=2) measuring 389 educators’ perception of their understanding related to physical literacy concepts; 3) 390 Attitude (n=3) measuring educators’ attitudes regarding physical literacy and health outcomes; 4) 391 Self Efficacy (n=3) measuring educator’s self-efficacy towards developing physical literacy 392 skills in the early learning environment; 5) Environment Behaviors (n=3) measuring educators’ 3 393 perception of their physical literacy promotion practices in the ECE environment; 6) 394 and Personal Behaviors (n=3) measuring perception of personal physical activity behaviors 395 (Table 6). This sentence is extremely long---I think it would be easier to read if they were separate sentences

463 they relate to health [17]. The PLKASB-ECE subscales of knowledge and perception of 464 knowledge address ~~with~~in this

473 competence required to participate in various activities [17]. We identified existing tools in the 474 literature targeting fundamental skill development for preschool (3-5 years) children that relate to 4 475 this physical competence[59–62] as this aspect of physical literacy was not included in the 476 PLKASB-ECE.

In the Strength and Limitations sections please take about the numbers
