# Supplementary material for: Validity and reliability of a physical literacy knowledge, attitudes, self-efficacy and behaviors questionnaire for early childhood educators (PLKASB-ECE): An exploratory factor analysis
Source: PLoS One. 2024 Oct 28;19(10):e0312736. doi: 10.1371/journal.pone.0312736 (PMC11516012; doi:10.1371/journal.pone.0312736)
Supplement: Attachment — Submitted filename: Response to Editors.docx [file pone.0312736.s006.docx]

PONE-D-24-06642R2

**Validity and reliability of a physical literacy knowledge, attitudes, self-efficacy and behaviors questionnaire for early childhood educators (PLKASB-ECE): An exploratory factor analysis**

PLOS ONE

**Journal Requirements**

**Journal Requirements #1**

Any changes to the reference list should be mentioned in the rebuttal letter that accompanies your revised manuscript.

**Author Response #1:** We have removed the following references from the revised manuscript in response to Editor Comment #3.

32.	Early childhood grows up: Towards a critical ecology of the profession: Setting the scene. Early Childhood Grows Up: Towards a Critical Ecology of the Profession. 2012. pp. 3–19. doi:10.1007/978-94-007-2718-2_1

33.	Ajzen I. The theory of planned behavior. Theor Cogn Self-Regul. 1991;50: 179–211. doi:10.1016/0749-5978(91)90020-T

36.	Janz NK, Becker MH. The Health Belief Model: A Decade Later. Health Educ Q. 1984;11: 1–47. doi:10.1177/109019818401100101

37.	Ajzen I, Fishbein M. Attitudes and the Attitude-Behavior Relation: Reasoned and Automatic Processes. Eur Rev Soc Psychol. 2000;11: 1–33. doi:10.1080/14792779943000116

38.	Bandura A. Social learning theory. Englewood Cliffs, N.J: Prentice Hall; 1977.

**Editor**

Editors - Thank you for all of the work on this mansucript. It is clearer and much easier to read. There is not a copy editor for the journal so I have identified a few very minor issues that will take you just a few minutes to correct. Thank yo very much

**Authors –** thank you very much for the opportunity and effort to make this a better paper. We are grateful for the review and comments.

**Editor Comment #1 -** Objective: The purpose of this study was to develop a holistic digital tool to measure physical literacy knowledge, attitudes, self-efficacy, and behaviors of early childhood educators (PLKASB-ECE) for both their professional teaching context as well as the educator’s own

**Author Response #1 –** Line #27 reflects this change

**Editor Comment #2 -** A 78 systematic review of objectively measured physical activity in center-based child care revealed that 79 physical inactivity levels are high across worldwide geographical locations [13]

**Author Response #2 –** Line #78 reflects this change

**Editor Comment #3 –** This section seems like a list of statements that is not supporting the paragraph it is in—please take it out

“*Exploring educator’s social cognitions can provide deeper insights 117 into how they can influence their behaviors in supporting the development of children’s physical 118 literacy. A complex set of factors govern educational decisions and behaviors within the ECEC 119 environment. The critical ecology framework [32] proposes that educator behaviors are 120 influenced by relationships and social interactions within the various contexts of an ECEC 121 environment. Social cognitive factors have been used to understand why people engage in certain 122 behaviors and are believed to predict intentional behavior [33–36]. Social cognitions include 123 constructs such as attitude and efficacy beliefs. Attitude is defined as the perceived favorable or 124 unfavorable evaluation of a certain behavior [37], while self-efficacy refers to the belief in one’s 125 ability to perform a particular behavior [38].”*

**Author Response #3 –** Line #116-125 have been removed. The reference list will reflect the deletion of these citations. Deleted citations include 32, 33, 36, 37, and 38.

**Editor Comment #4 –** Please make these into three separate sentences

*“Six were review articles [63–68], twelve addressed kindergarten to grade 6 [69–76] or 286 grade 7 to 12 [18,77–79] school aged children motor skills measurement of physical literacy, one 287 article described the development of a physical literacy questionnaire for primary school teachers 14 288 [80], and one provided a qualitative assessment of physical literacy in primary school teachers 289 [81].”*

**Author Response #4 –** This section has been revised. It now reads –

*“Six review articles [63–68] examined literature on physical literacy. Twelve articles focused on the measurement of motor skills related to physical literacy in school-aged children, specifically from kindergarten to grade 6 [69–76] and grades 7 to 12 [18,77–79]. Additionally, one article described the development of a physical literacy questionnaire for primary school teachers [80], and another provided a qualitative assessment of physical literacy among this group [81].”*

**Editor Comment #5 –** This sentence is extremely long---I think it would be easier to read if they were separate sentences

*“It consists of six subscales: 1) Knowledge sum score (n=7) 388 measuring educators’ physical literacy knowledge; 2) Perception of Knowledge (n=2) measuring 389 educators’ perception of their understanding related to physical literacy concepts; 3) 390 Attitude (n=3) measuring educators’ attitudes regarding physical literacy and health outcomes; 4) 391 Self Efficacy (n=3) measuring educator’s self-efficacy towards developing physical literacy 392 skills in the early learning environment; 5) Environment Behaviors (n=3) measuring educators’ 3 393 perception of their physical literacy promotion practices in the ECE environment; 6) 394 and Personal Behaviors (n=3) measuring perception of personal physical activity behaviors 395 (Table 6).”*

**Author Response #5 –** This section has been revised. It now reads –

*The instrument consists of six subscales. The first is the Knowledge Sum Score (n=7) measuring educators’ physical literacy knowledge by summing all correct answers for the score. The second subscale is* *the Perception of Knowledge (n=2) measuring educators’ perception of their understanding related to physical literacy concepts. The third subscale is Attitude (n=3) measuring educators’ attitudes regarding physical literacy and health outcomes. The fourth subscale is Self-Efficacy (n=3) measuring educator’s self-efficacy towards developing physical literacy skills in the early learning environment. The fifth subscale is Environment Behaviors (n=3) measuring educators’ perception of their physical literacy promotion practices in the ECE environment. The sixth subscale is Personal Behaviors (n=3) measuring perception of personal physical activity behaviors (Table 6).*

**Editor Comment #6 –**they relate to health [17]. The PLKASB-ECE subscales of knowledge and perception of knowledge address ~~with~~in this

**Author Response #6 –** Line #467 reflects this request. I have also changed the subscale capitalization in this paragraph to align with 388-397.

*The PLKASB-ECE subscales of Knowledge and Perception of Knowledge address ~~within~~ this domain. The affective domain refers to the motivation and confidence to incorporate movement into daily life [17]. The PLKASB-ECE subscales of Attitude and Self-Efficacy address belief that physical activity supports health as well as confidence in being able to include it within daily practice. As such these subscales fit within the affective domain. The behavioral domain addresses the engagement in physical activities on a daily basis [17]. The PLKASB-ECE subscales of Environmental Behavior and Personal Behavior speak to the educator actions related to taking responsibility for physical literacy experiences with their classroom setting as well as including physical activity in their personal daily practice which align with the behavioural domain.*

**Editor Comment #7 -**

473 competence required to participate in various activities [17]. We identified existing tools in the 474 literature targeting fundamental skill development for preschool (3-5 years) children that relate to 4 475 this physical competence [59–62] as this aspect of physical literacy was not included in the 476 PLKASB-ECE.

**Author Response #7 –** Line #478 reflects this change

**Editor Comment #8 -**

In the Strength and Limitations sections please take about the numbers

**Author Response #8 –** I believe this request pertains to the removal of numbers regarding the proportion of centers and women. These numbers have been removed from Line 510-515 and now reads:

*The proportion of recruited centers (large urban, medium, and small population centers) were relatively similar to overall population distribution across the province [50]. Some limitations should be considered when interpreting the findings of this study. First, the study sample population was limited to the province of Alberta, Canada which limits the generalizability of the findings. Second, most participants were women. However, this proportion is similar to the ECEC workforce in Canada so this sampling is reflective of the intended population [91].*
